# Comparative Analysis of Hematological and Immunological Parameters in Patients with Primary Sjögren’s Syndrome and Peripheral Neuropathy

**DOI:** 10.3390/jcm12113672

**Published:** 2023-05-25

**Authors:** Ancuta Mihai, Diana Maria Chitimus, Ciprian Jurcut, Florin Cristian Blajut, Daniela Opris-Belinski, Constantin Caruntu, Ruxandra Ionescu, Ana Caruntu

**Affiliations:** 1 Department of Internal Medicine, Carol Davila Central Military Emergency Hospital, 010825 Bucharest, Romania; coca.ancuta@gmail.com (A.M.); c.jurcut@gmail.com (C.J.); 2 Department of Rheumatology, Faculty of General Medicine, Titu Maiorescu University, 031593 Bucharest, Romania; 3 Department of Neurology, Carol Davila Central Military Emergency Hospital, 010825 Bucharest, Romania; chitimus.diana@yahoo.com; 4 Department of General Surgery, Carol Davila Central Military Emergency Hospital, 010825 Bucharest, Romania; cristi.blajut@gmail.com; 5 Department of Medical-Surgical Specialties, “Titu Maiorescu” University of Bucharest, 040441 Bucharest, Romania; 6 Internal Medicine and Rheumatology Department, Sfanta Maria Clinical Hospital, 011172 Bucharest, Romania; danaopris0103@yahoo.com (D.O.-B.); ruxandraionescu1@gmail.com (R.I.); 7 Internal Medicine and Rheumatology Department, Carol Davila University of Medicine and Pharmacy, 020021 Bucharest, Romania; 8 Department of Physiology, Carol Davila University of Medicine and Pharmacy, 020021 Bucharest, Romania; 9 Department of Dermatology, Prof. N.C. Paulescu National Institute of Diabetes, Nutrition and Metabolic Diseases, 011233 Bucharest, Romania; 10 Department of Oral and Maxillofacial Surgery, Carol Davila Central Military Emergency Hospital, 010825 Bucharest, Romania; ana.caruntu@gmail.com; 11 Department of Oral and Maxillofacial Surgery, Faculty of Dental Medicine, Titu Maiorescu University, 031593 Bucharest, Romania

**Keywords:** primary Sjögren syndrome, peripheral neuropathy, neutrophil to lymphocyte ratio, monocyte to lymphocyte ratio, gammaglobulins, C4, vitamin D

## Abstract

Background: Primary Sjögren syndrome (pSS) is a multisystem disorder of autoimmune etiology, frequently involving peripheral nerves. Early detection of peripheral neuropathy (PN) manifestations might improve prognosis and disease control. The purpose of the study was to evaluate the predictive potential of hematological and immunological parameters associated with PN development in pSS patients. Methods: This single-center retrospective study included patients with pSS who were divided into two groups, according to the occurrence of neurological manifestations throughout the follow-up period. Results: From the total of 121 pSS patients included in the study, 31 (25.61%) developed neurological manifestations (PN+ group) during the follow-up period. At the moment of pSS diagnosis, 80.64% of PN+ patients exhibited increased disease activity, with ESSDAI scores above 14 (*p* = 0.001), and significantly higher values for VASp score (*p* = 0.001), with a mean value of 4.90 ± 2.45, compared to 1.27 ± 1.32 in the PN- group. The hematological assessment at the moment of pSS diagnosis revealed that neutrophils and neutrophil-to-lymphocyte ratio (NLR) were significantly higher in the PN+ group (*p* = 0.001), while lymphocytes, monocytes and monocyte-to-lymphocyte ratio (MLR) were significantly lower (*p* = 0.025, *p* = 0.13 and *p* = 0.003, respectively). Immuno-inflammatory parameters—gammaglobulins, complement fractions C3, C4, total proteins and vitamin D were significantly lower in the PN+ patients’ group. In multivariate analysis, the independent predictive character for PN development in pSS patients was confirmed for NLR (95% CI 0.033 to 0.263, *p* = 0.012), MLR (95% CI −1.289 to −0.194, *p* = 0.008), gammaglobulins (95% CI −0.426 to −0.088, *p* < 0.003), complement fraction C4 (95% CI −0.018 to −0.001, *p* < 0.030) and vitamin D (95% CI −0.017 to −0.003, *p* < 0.009). Conclusions: Readily available and frequently used hematological and immunological markers, such as NLR, MLR, gammaglobulins, C4 and vitamin D could be helpful in predicting the neurological involvement in pSS patients. These biological parameters might become useful tools for clinicians to monitor disease progression and identify potentially severe extraglandular manifestations in pSS patients.

## 1. Introduction

Primary Sjögren’s syndrome (pSS) is a systemic autoimmune inflammatory disease which associates lymphocytic infiltration of exocrine glands [1]. Many pSS patients may experience extraglandular manifestations, such as cardiac, joint, gastroenterology, skin, pulmonary, kidney and neurological involvement [2,3,4].

Neurological manifestations may affect the central and peripheral nervous system, with a prevalence between 5.3% and 21% [5,6]. Central nervous system (CNS) involvement is a rare complication of pSS; contrarily, peripheral nervous system (PNS) manifestations seem to be more common. Furthermore, peripheral neuropathy (PN) is the presenting manifestation in a quarter of pSS patients [7]. The most common PN form is distal sensory axonal polyneuropathy, followed by sensorimotor neuropathy [8]. Sensory axonal neuropathy, known as pure sensory neuropathy (PSN), usually affects the lower limbs and manifests as distal paresthesias and sensitive impairment, with symmetrical character, that can be accompanied by burning feet pain [9,10]. Sensorimotor polyneuropathy (SMN) occurs when motor nerve fibers are involved, clinically expressed as muscle weakness, in addition to sensory axonal polyneuropathy [11]. The PN type is diagnosed by clinical neurological symptoms and signs, electromyographic assessment and nerve biopsy. Pseudo blocks corresponding to the areas of nerve ischemia are the distinctive signs detected in the electrophysiological investigation. Intriguingly, pSS is one of the most frequent causes of PSN among all immune-mediated diseases [12,13].

The role of hematological and immunological parameters in the assessment of autoimmune disease activity and follow-up is of great interest. Circulating cells, lymphocytes, monocytes, neutrophils and platelets play major roles in inflammatory processes, including neurological involvement [14,15,16]. Their ratios, neutrophil-to-lymphocyte ratio (NLR), monocyte-to-lymphocyte ratio (MLR) and other different ratios were studied in a wide range of diseases [17,18,19]. NLR and MLR are considered systemic parameters of inflammation and were correlated with increased disease activity in autoimmune disorders, such as systemic lupus erythematosus (SLE), rheumatoid arthritis (RA) or with neurological involvement, such as Guillain-Barré syndrome [20,21,22]. Moreover, immunological markers, traditionally used in pSS diagnosis and monitoring, such as gammaglobulins, complement fractions or vitamin D, exhibit significant changes in autoimmune diseases, suggesting a possible link in the disease pathogenesis [23,24]. Previous studies revealed that low levels of C3 and C4 complement fractions were correlated with systemic manifestations and disease activity in patients with SLE, vasculitis and myasthenia gravis [25,26]. In pSS, hypocomplementemia, C3 and C4 were correlated with extraglandular manifestations and disease activity [27]. High levels of gammaglobulins were associated with increased immune activity in many autoimmune diseases, such as SLE, lupus nephritis, autoimmune hepatitis and pSS [28,29]. The protective effect of vitamin D against autoimmune activity is believed to be exerted through B cells activation and differentiation mechanisms, with reduced immunoglobulin production, including autoantibodies [30,31,32]. In patients with vitamin D deficiency, increased inflammatory cytokine levels were detected, confirming its role as an immunomodulatory molecule [33]. Furthermore, reduced concentrations of vitamin D were associated with increased inflammation and high disease activity in SLE, intestinal bowel disease, RA and pSS [24].

This study aimed to evaluate hematologic and immunologic parameters and their predictive potential for PN development in pSS patients. This is a continuation of a previous study of our research group, which showed the predictive character of specific hematological parameters for the development of cutaneous vasculitis lesions in pSS patients [34]. The identification of accessible disease-predictive parameters in pSS could provide a timely selection of patients at risk for progression towards a severe disease, thus allowing a more efficient therapeutic management in pSS.

## 2. Materials and Methods

### 2.1. Study Design and Data Collection

We retrospectively analyzed the medical records of pSS patients admitted to our department between April 2015 and December 2021. All patients were diagnosed according to the American European Consensus Group criteria (AECG) [35] and ACR/EULAR classification criteria for pSS [36]. Only newly diagnosed patients with pSS who did not receive any previous disease-specific treatment were included in the study. In pSS patients with negative serum antibodies, a biopsy of the minor salivary glands was performed, in order to validate the diagnosis. Patients with pre-existing neurological diseases, including pSS patients with neurological involvement at the moment of diagnosis, diabetic, toxic, metabolic, amyloid, as well as paraneoplastic neuropathy, other autoimmune diseases, lymphoproliferative disorders and active infections, were excluded from the study. Moreover, patients with gastrointestinal disease or malabsorption conditions were also excluded.

For every pSS patient, glandular and extra-glandular disease manifestations were recorded at the initial visit. All newly diagnosed patients were enrolled in a periodical follow-up program, with trimestral visits, to reassess disease progression, including the occurrence of extraglandular manifestations, and the response to therapy. The general assessment of the patients involved blood and urinary tests, electrocardiogram and ultrasound of salivary glands and abdomen. Patients who expressed neurological complaints, such as painful or burning paraesthesias involving proximal parts of limbs, trunk or face paresis, gait disturbance, muscle cramps and bladder dysfunction were clinically examined by neurologists, additionally using nerve conduction studies and electrochemical skin conductance. A reduced version of the total neuropathy score (TNSr) was used to evaluated the severity of neuropathy. The TNSr scale includes assessments of sensory, motor and autonomic symptoms; pin sensibility; strength evaluation; tendon reflexes and vibration sensibility [37]. The score values were divided as follows: score 0 indicates no PN, scores between 1 and 9 indicate mild PN, scores between 10 and 19 indicate moderate PN, and a score > 20 corresponds to severe PN, as documented in previous studies on pSS-associated PN [38]. We also performed the electrophysiological examination that included sensory nerve action potential (SNAP), nerve conduction velocities, compound muscle action potential (CMAP), and F-wave in the ulnar, median, tibial, peroneal and sural nerves [39]. Needle electromyography was also performed to exclude the possibility of any muscle pathologies [40]. Electrochemical skin conductance was evaluated in patients with symptoms such as tingling, pain and loss of temperature detection, using SUDOSCAN [41]. The normal sudomotor function had an interval of 60–100 µS, moderately reduced sudomotor function: 40–60 µS, while severely reduced function: 0–40 µS. Evaluation for pain was carried out using a visual analogue scale (VASp) [42], with a 0–100 mm score.

For analysis of complete blood count and inflammatory and immunological parameters, blood samples were collected between 7 a.m. and 11 a.m. for all patients. Disease activity was evaluated using the ESSDAI score [35], which included both clinical and biological domains. For patients with mild disease activity (ESSDAI score 5–13), conservatory treatment was recommended, such as hygienic-dietary and topical treatments, while for patients with moderate to severe disease activity (ESSDAI score ≥ 14), systemic therapies were prescribed. Nerve biopsy was not performed in any of the patients included in the PN+ group.

The treatment of neurological involvement in pSS patients included corticosteroids, hydroxychloroquine, azathioprine and cyclophosphamide. In refractory cases of patients, plasmapheresis, intravenous immunoglobulins (IVIg) and rituximab were efficient treatments [43].

The research was approved by the hospital’s Ethics Committee (No 365/11.02.2020), and all patients signed informed consent before the inclusion of their information in the study.

### 2.2. Data Collection

Data of pSS patients included in this study were extracted from the patient’s admission charts and the hospital’s database. Hematological, immunological and inflammatory markers determined at disease diagnosis were recorded for this study, namely complete blood count (CBC) and derived hematological ratios, NLR, platelets-to-lymphocyte ratio (PLR), MLR, erythrocyte sedimentation rate (ESR), hs-C reactive protein (hs-CRP), complement C3 and C4 levels, immunoglobulins (IgA, IgM, IgG), rheumatoid factor (RF), proteins electrophoresis, antinuclear antibodies (ANA), anti-Ro/SSA and anti-La/SSB antibodies, serum cryoglobulins and vitamin D. Peripheral blood samples of the pSS patients were analyzed according to current medical standards. CBC was collected in dipotassium ethylenediaminetetraacetic acid (EDTA) tubes (Vacuette, Greiner Bio-One, Frickenhausen, Germany) using fluorescence flow cytometry technology and was analyzed using Sysmex XN-3000 (Sysmex Corporation, Kobe, Japan). For immunological parameters, immunoturbidimetry was used, and the samples were analyzed by Beckman Coulter AU5812 (Beckman Coulter, Inc., Brea, CA, USA). Gammaglobulins were determined by migration in the electric field using Sebia Capillarys Sebia 3 OCTA (Sebia, Lisses, France). The levels of ANA, anti-Ro/SSA and anti-La/SSB antibodies were determined by enzyme-linked immunosorbent assay (ELISA) technology using ORGENTEC Alegria 2 analyzer (ORGENTEC Diagnostika GmbH, Mainz, Germany). Vitamin D was determined by chemiluminescent microparticle immunoassay (CIMA) and analyzed by ORGENTEC Alegria 2 analyzer (ORGENTEC Diagnostika GmbH, Mainz, Germany). Vitamin D deficiency was defined by plasma levels <20 ng/mL [44].

Nerve conduction studies were performed using the Nihon Kochden electromyography machine, while the electrochemical skin conductance was evaluated with SUDOSCAN (Impeto Medical, Paris, France) [41,45].

### 2.3. Statistical Analysis

Database management and statistical analyses were performed using SPSS software (version 26.0, SPSS, Chicago, IL, USA). The distribution normality was determined by the Kolmogorov–Smirnov test and the Shapiro–Wilk test. In order to evaluate the differences between groups, parametric or non-parametric tests were used according to the distribution pattern. Furthermore, the sensitivity and specificity of the investigated parameters were determined using ROC curve analysis, and the cut-off values were calculated using the Youden index. The parameters that showed significant differences between the two groups were subsequently tested by multiple linear regression analysis. Continuous variables were presented as mean ± standard deviation while categorical variables were indicated as a percentage. Two-tailed *p*-values of less than 0.05 were considered to indicate statistical significance.

## 3. Results

### 3.1. General Characteristics of the Study Groups

From 131 patients diagnosed with pSS in our department between 2015 and 2021, 121 patients met the eligibility criteria and were included in our study. The mean age of the study group was 49.65 ± 10.75 (range: 19–68) and the general gender distribution was 118 (97%) females and 3 (3%) males. During the follow-up interval, 31 pSS patients, representing 26% of the study group, developed PN. The mean follow-up period for the PN+ group was 58.65 ± 19.64 months, while for the PN- group, it was 60.15 ± 13.29 months. The comparative analysis of the follow-up period between the two groups revealed no significant differences. Moreover, no differences were detected in the gender distribution and mean age values between the patients who developed PN and the rest of the patients (49.65 ± 10.75, 47.03 ± 11.79, respectively). Results are shown in Table 1.

While xerostomia was the main complaint in the vast majority of the patients, without correlation with the subsequent development of PN (90% of PN− group and 92% of PN+ group), xerophtalmia was confirmed at diagnosis in a significantly higher number of patients who later developed PN (*p* = 0.003). Thus, in 84% of patients from the PN+ group, xerophtalmia was clinically expressed at disease diagnosis, compared to only 53% of patients from the PN− group.

The analysis of disease activity scores at baseline visit revealed significant differences between pSS patients who developed PN during the follow-up period and the PN− group. Thus, patients from the PN+ group had significantly higher VASp scores at the initial visit, with a mean value of 4.90 ± 2.45, compared to the patients from the PN- group, in which the mean VASp value was 1.27 ± 1.32 (*p* = 0.001). Similarly, the mean baseline value for ESSDAI score was significantly higher in the PN+ group of patients compared to the PN− group (*p* = 0.001). Most of the patients from PN+ group (81%) had a baseline ESSDAI score ≥ 14 (*p* = 0.001), while a similar score was found in only half of the patients from the PN− group (51%). Results are presented in Table 1.

### 3.2. Comparative Analysis of the Laboratory Parameters

The pSS patients who developed PN during the follow-up period revealed significantly higher values for neutrophils at the initial visit compared to patients without PN (*p* = 0.001), while the mean values for lymphocytes and monocyte were significantly lower (*p* = 0.025, *p* = 0.013, respectively). The analysis of cellular ratios revealed that NLR was significantly higher in the PN+ group of patients, with a mean value of 3.14 ± 0.76, compared to 2.58 ± 0.58 in the PN− group (*p* = 0.001), while MLR was significantly lower in the PN+ group, with a mean value of 0.24 ± 0.96, compared to 0.30 ± 0.12 in the PN− group (*p* = 0.003). The comparative analysis of pSS-specific immunological parameters revealed a significantly lower baseline mean value for total proteins and gammaglobulins in the PN+ group of patients, compared to PN− group (*p* = 0.019, *p* = 0.012, respectively). A similar trend was found for the complement fractions, C3 and C4, which had significantly lower values in the PN+ group compared to PN− group (*p* = 0.022, *p* = 0.001, respectively). The determination for vitamin D at the moment of pSS diagnosis revealed significantly lower levels in the PN+ group of patients, with a mean value of 13.90 ± 7.19, compared to PN− group of patients, in which the mean value was 24.86 ± 11.04 (*p* = 0.001). No differences were detected in patients who subsequently developed PN from the rest of the patients for ANA, anti-Ro/SSA, anti-La/SSB antibodies, RF, immunoglobulins and inflammatory markers (ESR, hs-CRP, cryoglobulins). The results are presented in Table 2.

### 3.3. Investigation of PN and Other Extraglandular Manifestations

During the follow-up period, about a quarter of patients (26%) developed PN. The neurological investigation revealed that in 21 patients, representing two thirds of the PN+ group, the clinical expression was of PSN, the most frequent PN form, while SMN was detected in 10 patients.

The application of TNSr scale revealed that most of the patients, 81%, presented moderate neuropathy, while severe neuropathy was reported in 19%. Skin conductance investigated with SUDOSCAN revealed that two thirds of the patients developed moderate or severe disfunction.

Among the extraglandular manifestations developed during the follow-up period, purpura was diagnosed with a similar incidence in both patients from the PN+ group (26%) and patients from the PN- group (27%), without any significant differences (*p* = 0.564). Similarly, two patients from the PN+ group (6%) developed non-Hodgkin lymphoma with B cells (B-NHL), without reaching the threshold for statistical significance (*p* = 0.064). The results are presented in Table 3. Other extraglandular manifestations confirmed in our pSS patients at baseline revealed no significant differences between PN+ and PN- groups of patients, except for renal involvement that was more common at baseline in pSS patients who subsequently developed neurologic manifestations (Table S1).

### 3.4. Receiver-Operating Characteristic (ROC) Curves for NLR, MLR, Total Proteins, Gammaglobulins, C3 and C4, and Vitamin D for the Prediction of Neurological Involvement in pSS Patients

**Figure 1:**
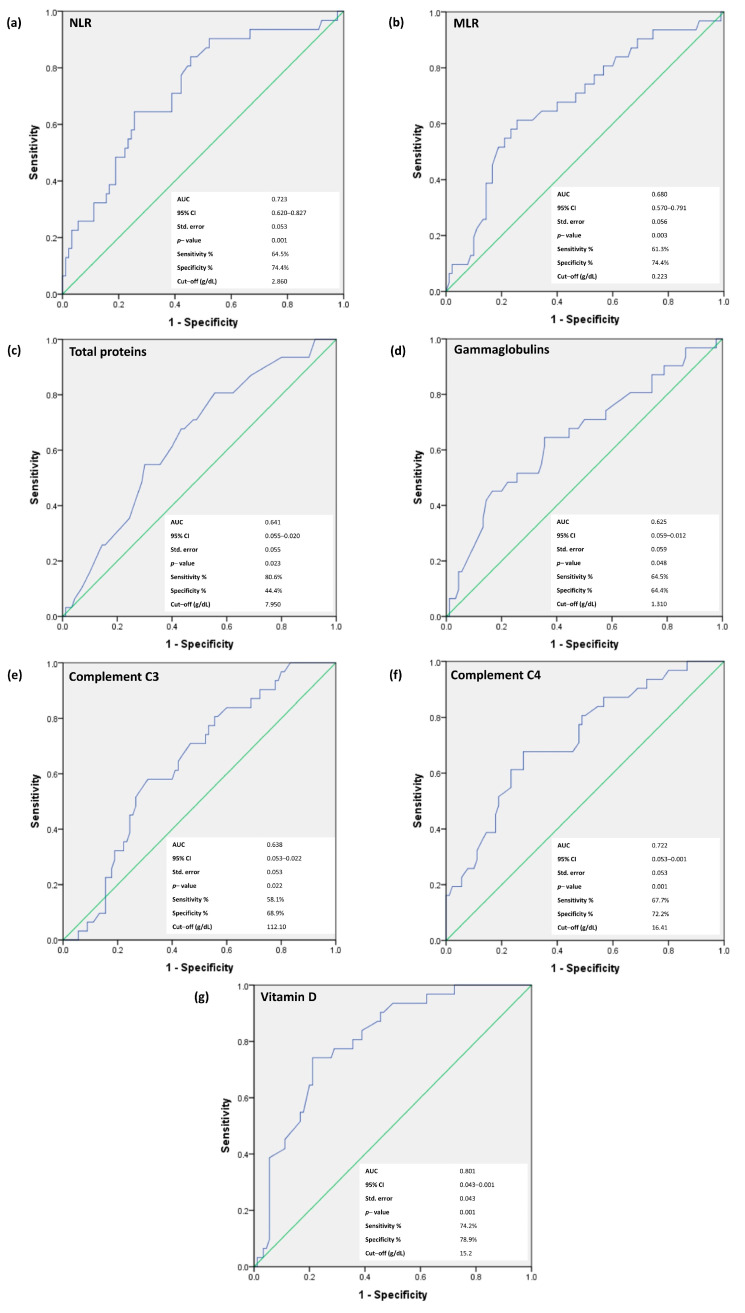
Receiver operating characteristic curve of (**a**) NLR, (**b**) MLR, (**c**) total proteins, (**d**) gammaglobulins, (**e**) C3, (**f**) C4 and (**g**) vitamin D levels for the prediction of the neurological involvement in pSS patients.

We continued the analysis focusing on NLR, MLR, total proteins, gammaglobulins, C3 and C4 and vitamin D—all parameters that revealed statistically significant differences in patients who developed PN during the follow-up period. We excluded the individual cell counts from this analysis, because they were enclosed in the cellular ratios—NLR and MLR. The ROC curve analysis was used for the prediction of neurological involvement in pSS patients in order to determine the optimal threshold for the variables, maximizing the composite of specificity and sensitivity (Figure 1).

In the prediction of PN development in pSS patients, baseline vitamin D levels revealed the strongest statistical power correlations. Thus, for vitamin D, the area under the curve (AUC) was 0.801 (*p* < 0.001), with a sensitivity of 74.2% and a specificity of 78.9%, for the cut-off value of 15.2 ng/mL. In addition, the analysis of cellular ratios revealed that for NLR, the AUC was 0.723 (*p* = 0.001), with a sensitivity of 64.5% and a specificity of 74.4%, while for MLR, the AUC was 0.680 (*p* = 0.003), with a sensitivity of 61.3% and a specificity of 74.4%. The cut-off values for these parameters were 2.860 for NLR and 0.223 for MLR. For total proteins, the AUC was 0.641 (*p* = 0.023), with a sensitivity of 80.6% and a specificity of 44.4%, and for gammaglobulins, the AUC was 0.625 (*p* = 0.048), with a sensitivity of 64.5% and a specificity of 64.4%. The cut-off values were 7.950 g/dL for total proteins and 1.310 g/dL for gammaglobulins. ROC curve analysis for complement fraction C3 was expressed with an AUC of 0.638 (*p* < 0.022), with a sensitivity of 58.1% and a specificity of 68.9%, and the cut-off value of 112.10 mg/dL, while for complement C4, the AUC area was 0.722 (*p* = 0.001), with a sensitivity of 67.7% and a specificity of 72.2% and an optimized cut-off value of 16.41 mg/mL.

In the multivariate analysis, performed with multiple linear regression, the most relevant hematological and immunological parameters were considered elements that might predict the development of PN in pSS patients. The ESSDAI score was excluded from this analysis because it includes within the scale the dependable variable. Similarly, the gender of patients was excluded due to the discrepancy in the male and female ratio (3 males and 118 females). The independent prediction character for the development of PN in pSS patients was confirmed for NLR (95% CI 0.033 to 0.263, *p* < 0.012) and MLR (95% CI −1.289 to −0.194, *p* < 0.008). It was also confirmed for gammaglobulins (95% CI −0.426 to −0.088, *p* < 0.003), complement C4 (95% CI −0.018 to −0.001, *p* < 0.030) and vitamin D (95% CI −0.017 to −0.003, *p* < 0.009) (Table 4). Total proteins and complement C3 did not reach the statistical significance threshold in the multivariate analysis (*p* < 0.081, respectively, *p* < 0.847). The R square for this model was 0.383 (*p* = 0.001).

## 4. Discussion

Extraglandular manifestations (EM) of pSS play a significant role in the long-term prognosis of the disease [23]. An early diagnosis of EM through accessible and inexpensive tests could positively impact the disease progression and the quality of life for pSS patients. We aimed to investigate the prognostic potential of different hematological and immunological parameters for the development of peripheral neuropathy in pSS. Previous studies showed that neurological involvement in pSS was associated with increased severity of the disease and with multiple other extraglandular manifestations [23].

Our analysis revealed significant differences in the routine blood tests, detectable from the moment of pSS diagnosis, in patients who subsequently developed PN during the follow-up period. Higher neutrophil counts in peripheral blood were found in these patients in comparison to pSS patients without neurological involvement. Previous studies indicated that neutrophils promote inflammation and contribute to the pathogenesis of autoimmune diseases, such as rheumatoid arthritis (RA), multiple sclerosis (MS), systemic lupus erythematosus (SLE), Crohn’s disease (CD) and pSS [46]. Rapid activation and an exponential increase in their lifespan led to their long-term existence at the inflammation site [46]. It seems that neutrophils act as immune cells in autoimmune diseases, their infiltration being achieved through complex immune mechanisms and complement system-mediated processes [47]. By contrast, our investigation revealed significantly lower baseline values for lymphocyte and monocyte counts in peripheral blood in pSS patients who subsequently developed neurological manifestations. In autoimmune diseases, an intense tissular infiltration with lymphocytes was reported, which could lead to a reduced number of circulating cells [22]. Lymphocytes are essential players in autoimmune diseases, and lymphocytic infiltration was reported in all the tissues with chronic inflammation [48]. In pSS, lymphocytes participate in the intratissular production of auto-antibodies and were associated with an increased risk of lymphoma development due to chronic antigenic stimulation [49,50]. Monocytes, important elements of the innate immunity, serve as a connection to the adaptive immune system [51], through the release of a large range of cytokines and chemokines with pro-inflammatory, proliferative and regulatory functions [52]. In autoimmune diseases, low circulating levels of monocytes are attributed to their recruitment in the affected tissues [53,54].

The analysis of cellular ratios revealed higher values for NLR and lower values for MLR in pSS patients who developed neurological manifestations during the follow-up period, as an expression of the balance between the components of these ratios, suggesting that the important drop in circulating lymphocytes count is the main actor in influencing these changes. In different autoimmune diseases, such as SLE, RA, systemic sclerosis (SSc), polymyositis/dermatomyositis (PM/DM) and pSS, higher values of hematological ratios, NLR and MLR, positively correlated with inflammatory markers and disease activity, [22,55]. Furthermore, NLR revealed significant correlation with peripheral neuropathy in diabetic patients, suggesting that it may be used as an independent prognostic parameter in this disease [56]. In pSS patients, increased NLR values were associated with higher ESSDAI scores, which positively correlated with neurological involvement [55]. Different types of inflammatory cells were detected in the perineural infiltrate in the context of neurological manifestations. In peripheral neuropathy, a perivascular and endoneurial lymphocytic infiltration of the nerves was described, which leads to demyelination, axonal damage and the progressive loss of motor functions [57,58]. Furthermore, lymphocytes release pro-inflammatory cytokines that can activate nociceptive nerve terminals, leading to nociceptor sensitization and neuropathic pain. A rich neutrophilic infiltrate surrounding the injured nerves was also shown to contribute to nociception [57], while perineural monocytic infiltration can orchestrate the emergence of mechanical hypersensitivity, leading to a variety of peripheral neurological expressions [54,59]. The ROC curve analysis revealed the superior predictive power of NLR compared to MLR, with a higher sensitivity at the determined cut-off value, for the early detection of neurological involvement in pSS patients.

Significantly lower baseline values for gammaglobulins and complement fraction C4 were registered in the PN+ group of patients. Furthermore, the sensitivity and specificity for PN prediction were higher for C4 fraction compared to gammaglobulins, emphasizing its superior power in the detection of pSS patients at risk for neurological impairment. Hypogammaglobulinemia was reported in different autoimmune diseases, such as SLE and pSS, having various etiologies [60]. The complement system is considered to play an important role in increasing antibody production, clearance of immune complexes and cell lysis, while an impaired clearance of cellular debris may facilitate the exposure of various cellular self-antigens followed by loss of tolerance [26,61]. The complement fixation and consumption may play an essential role in neurologic acute pathophysiology and facilitates neuroinflammation in autoimmune diseases [62,63]. Our findings are supported by previous studies which detected low complement C4 levels in pSS patients with peripheral neuropathy [23,64]. We found no significant differences between groups regarding the autoantibodies—ANA, anti-La/SSB and anti-Ro/SSA; these results were supported by previously reported data [23,65].

At the time of disease diagnosis, lower levels of vitamin D were detected in pSS patients who subsequently developed neurological manifestations. Vitamin D, besides its role in regulating bone metabolism and calcium homeostasis, is involved in the modulation of both adaptive and innate immune responses and in the maintenance of the balance between pro- and anti-inflammatory processes [66,67]. Thus, through vitamin D-mediated mechanisms, this impaired balance associated with autoimmune activity could be restored [68]. Moreover, vitamin D is involved in various neurological functions, such as neuroplasticity, neuroprotection and neurotransmission, and also has immunomodulatory effects inside the nervous system. The biosynthesis of neurotrophic factors, the synthesis of inducible nitric oxide synthase and the increase in glutathione levels are reported as biological effects of vitamin D on the nervous system [69]. The connection between vitamin D deficiency and peripheral neuropathy development in patients with diabetes, rheumatoid arthritis, SLE [70,71] and pSS were previously suggested [32]. In our group of patients, the lower levels of vitamin D detected at the time of pSS diagnosis could be a predisposing factor for secondary neurological complications. Similar findings were reported in other studies investing the role of vitamin D in the development of neuropathy [32]. Furthermore, the calculated cut-off value predictive for PN development fell into the range of vitamin D deficit. Thus, vitamin D levels should be assessed in all pSS patients and supplements should be administered whenever a deficit is detected, combined with the close monitoring for neurologic complications. Taking into consideration the calculated cut-off values for the independent predictive parameters for neurological involvement reported in our study, further studies could be conducted to determine the long term clinical relevance of these elements in the management of pSS patients. The analysis of the clinical characteristics in our pSS patients revealed that in patients who developed neurological manifestations during the follow-up period, an increased disease activity was detected even from the first presentation. Thus, ESSDAI and VASp scores were significantly higher in these patients compared to those without further neurological involvement. Xerostomia and xerophthalmia—the diagnostic criteria in pSS—were confirmed in most of our patients, with a significantly higher incidence of xerophthalmia as an initial manifestation in patients who subsequently developed neurological manifestations; similar findings were reported in previous studies [72]. During the follow-up period, alongside the neurological manifestations, cutaneous purpura occurred with a similar incidence in both groups of patients, while lymphoma was diagnosed exclusively in pSS patients who also developed neurological manifestations. In previous pSS studies, cutaneous purpura was reported with a high incidence both when diagnosed as a single entity or in association with neurological manifestations [23]. Lymphoma, a rare complication in pSS, was associated with neurological manifestations [73]. Furthermore, previous studies suggested that neurological manifestations in pSS patients may announce the upcoming development of lymphoma [74].

Peripheral neurological involvement in pSS can be defined by various expressions, such as distal axonal sensory poly-neuropathy, small fiber neuropathy, sensorimotor polyneuropathy, multiple mononeuropathies, all described as possible manifestations in different autoimmune diseases [75]. In our pSS patients, two forms of peripheral neuropathy were reported, PSN and SMN, with an important predominance of PSN, trends which are comparable to previously reported data [23,73,76]. The pathophysiology of neurological manifestations in pSS is not entirely understood. Different mechanisms were suggested, based on the histological and serological findings, such as vasculitis of the vasa nervorum with concomitant lymphocytic and macrophage infiltration, necrotizing vasculitis and anti-neuronal antibodies, according to the type of nerve involved [77,78]. Vasculitis of the vasa nervorum seems to be the main pathogenic mechanism in PSN and SMN [79]. Previous studies mentioned a perineurial lymphoplasmocytic infiltration observed on nerve biopsies of pSS patients with SMN, alongside an increased prevalence of cryoglobulins, a mechanism that may play a leading role in the pathogenesis of vasculitis [60]. The TNSr score revealed that all pSS patients with neurological manifestations exhibited moderate to severe impairment, while skin conductance, tested with SUDOSCAN, exhibited moderate and severe disfunction in most of these patients. Similar results were reported in previous studies [80,81]. These tests are also useful in monitoring the disease progression and the response to therapy in different pathologies, including pSS [80,82,83]. The diagnosis of EM depends on the multidisciplinary collaboration between rheumatologists, neurologists and internal medicine specialists. Globally accessible serum elements that would facilitate the identification of high-risk patients for neurological involvement in a timely manner might be very useful clinical instruments in the management of this complication in pSS. The limitations of the current study are represented by the single-center and retrospective character, as well as by the relatively small sample size; but, at the same time, comparable to other studies. In addition, follow up data and response to therapy in pSS patients with neurological involvement were not considered in our study. Therefore, multicenter, prospective studies on large cohorts of patients are necessary to further validate our findings on the predictive role of hematologic parameters in the neurological involvement of pSS patients, and to refine the optimized cut-off values for the assessed parameters.

## 5. Conclusions

The present study reported a correlation between changes in the hematological and immunological parameters in pSS and the subsequent development of peripheral neuropathy. NLR, MLR, gammaglobulins, C4 and vitamin D revealed statistically significant variations, even from the moment of pSS diagnosis, in patients who developed peripheral neuropathy during the follow-up period. Our results show that these cost-effective, reliable and widely available biomarkers may be potentially useful elements for the early detection of patients at risk for neurological manifestations. Furthermore, these biological parameters might become useful tools for clinicians to monitor disease progression and identify potentially severe extraglandular manifestations in pSS patients.

## Figures and Tables

**Table 1:** General characteristics and comparative analysis of the study groups.

	PN+ Group	PN− Group	*p*-Value
	Mean ± SD	Mean ± SD	
	No = 31 (25.61%)	No = 90 (74.38%)	
**Demographic Characteristics**			
Age at pSS diagnosis (years)	49.65 ± 10.75	47.03 ± 11.79	0.810 ^a^
Sex			0.569 ^a^
Female (*n*, %) Male (*n*, %)	31 (100%)	87 (96.66%)	-
	-	3 (3.33%)	-
**Glandular Manifestations**			
Xerostomia, *n* (%)	28 (90.32%)	83 (92.22%)	0.497 ^a^
Xerophthalmia, *n* (%)	26 (83.87%)	48 (53.33%)	**0.003 ^a^**
**Clinical Scores**			
VAS pain score (mm)	4.90 ± 2.45	1.27 ± 1.32	**0.001 ^b^**
ESSDAI score	20.35 ± 7.56	15.13 ± 7.57	**0.001 ^b^**
ESSDAI score 5–13	6 (19.35%)	44 (48.88%)	**0.001 ^a^**
ESSDAI score ≥ 14	25 (80.64%)	46 (51.11%)	

Abbreviations: PN, peripheral neuropathy; VAS, visual analog scale; ESSDAI, EULAR Sjögren’s Syndrome Disease Activity Index; SD, standard deviation. ^a^ Chi- Square; ^b^ Mann–Whitney U test; statistical significance < 0.05.

**Table 2:** Hematological and immunological features of pSS patients from the PN+ and PN− groups.

Laboratory Findings	PN+ Group Mean ± SD	PN− Group Mean ± SD	*p*-Value
**Cells Count**			
Neutrophils (10^9^/µL)	3.42 ± 0.83	2.89 ± 0.53	**0.001 ^b^**
Lymphocytes (10^9^/µL)	1.09 ± 0.99	1.13 ± 0.09	**0.025 ^b^**
Monocyte (10^9^/µL)	0.25 ± 0.70	0.34 ± 0.14	**0.013 ^b^**
Platelets (10^9^/L)	262.65 ± 38.45	254.9 ± 54.25	0.131 ^b^
**Cellular Ratios**			
NLR	3.14 ± 0.76	2.58 ± 0.58	**0.001 ^b^**
MLR	0.24 ± 0.96	0.30 ± 0.12	**0.003 ^b^**
PLR	241.58 ± 37.89	228.33 ± 58.72	0.056 ^b^
**Immunological Results**			
ANA (U/mL)	3.48 ± 2.21	4.23 ± 2.21	0.064 ^b^
positive (*n*, %)	26 (83.87%)	77 (85.55%)	0.777 ^a^
Anti-Ro/SSA (U/mL)	122.50 ± 85.58	145.76 ± 79.52	0.183 ^b^
positive (*n*, %)	23 (74.19%)	79 (87.77%)	0.089 ^a^
Anti-La/SSB (U/mL)	89.59 ± 138.60	91.2 ± 85.99	0.303 ^b^
positive (*n*, %)	17 (54.83%)	55 (61.11%)	0.672 ^a^
Total proteins (g/dL)	7.37 ± 0.68	7.76 ± 0.91	**0.019 ^b^**
Gammaglobulins (g/dL)	1.28 ± 0.39	1.48 ± 0.4	**0.012 ^b^**
IgA (mg/L)	248.39 ± 124.28	304.26 ± 160.37	0.054 ^b^
IgG (mg/L)	1514.19 ± 646.78	1797.25 ± 740.09	0.913 ^b^
IgM (mg/L)	175.03 ± 107.73	180.28 ± 131.85	0.525 ^b^
RF (U/mL)	54.67 ± 72.92	54.61 ± 59.98	0.529 ^b^
C3 (mg/dL)	113.94 ± 16.26	126.78 ± 38.72	**0.022 ^b^**
C4 (mg/dL)	16.17 ± 7.59	22.97 ± 8.56	**0.001 ^b^**
Vitamin D (ng/mL)	13.90 ± 7.19	24.86 ± 11.04	**0.001 ^b^**
**Inflammatory Results**			
ESR (mm/h)	33.81 ± 25.81	35.48 ± 24.46	0.563 ^b^
hs- CRP (mg/L)	27.22 ± 18.21	26.06 ± 30.32	0.263 ^b^
Cryoglobulins, *n* (%)	2 (6.45%)	6 (6.66%)	0.665 ^a^

Abbreviations: PN, peripheral neuropathy; MLR, monocyte to lymphocyte ratio; NLR, neutrophil to lymphocyte ratio; PLR, platelet to lymphocyte ratio, RF, rheumatoid factor; C3 and C4, complement 3 and 4; ESR, erythrocyte sedimentation rate; hs- CRP, high sensitivity C-reactive protein; ANA, antinuclear antibodies; SD, standard deviation. ^a^ Chi-Square; ^b^ Mann–Whitney U test; statistical significance < 0.05.

**Table 3:** Extraglandular manifestations, neuropathy score and skin conductance in pSS patients.

	PN+ Group	PN− Group	*p*-Value
	**Peripheral Neuropathy**		
Pure sensory neuropathy	21 (67.74%)	-	-
Sensorimotor polyneuropathy	10 (32.25%)	-	-
**Clinical Score**			
**TNSr**	14.65 ± 3.99	-	-
moderate neuropathy	25 (80.64%)		
severe neuropathy	6 (19.35%)		
**Sudoscan**	18 (43.90%)	-	-
no dysfunction	11 (35.48%)	-	-
moderate dysfunction	14 (45.16%)	-	-
severe dysfunction	6 (19.35%)	-	-
**Extraglandular Manifestation**			
Purpura, *n* (%)	8 (25.80%)	24 (26.66%)	0.564 ^a^
B-NHL, *n* (%)	2 (4.87%)	-	0.064 ^a^

Abbreviations: PN, peripheral neuropathy; B-NHL, non-Hodgkin lymphoma with B cell; TNSr, Total Neuropathy Score reduced. Score 0 indicates no PN, scores 1–9 indicate mild PN, scores 10–19 indicate moderate PN, score > 20 corresponds to severe PN. Sudoscan-Skin conductance is measured microSiemens (µS). Normal sudomotor function corresponds to an interval of 60–100 µS, moderately reduced sudomotor function: 40–60 µS, and severely reduced sudomotor function: 0–40 µS. ^a^ Chi- Square; statistical significance < 0.05.

**Table 4:** Multiple linear regression analysis for the development of PN in pSS patients.

Laboratory Findings	Unstandardized Coefficients		Standardized Coefficients	T	*p*	95.0% Confidence Interval for B	
	Β	Standard Error	Beta			Lower Bound	Upper Bound
(Constant)	**0.387**	0.393	-	3.527	0.001 *	0.608	2.166
NLR	0.148	0.058	0.230	2.542	0.012 *	0.033	0.263
MLR	−0.741	0.276	−0.211	−2.680	0.008 *	−1.289	−0.194
Total proteins (g/dL)	−0.066	0.038	−0.133	−1.759	0.081	−0.141	0.008
Gammaglobulins (g/dL)	−0.257	0.085	−0.238	−3.013	0.003 *	−0.426	−0.088
C3 (mg/dL)	0.001	0.001	−0.016	−0.194	0.847	−0.002	0.002
C4 (mg/dL)	−0.009	0.004	−0.186	−2.204	0.030 *	−0.018	−0.001
Vitamin D (ng/mL)	−0.010	0.004	−0.257	−2.657	0.009 *	−0.017	−0.003
Age	0.003	0.003	0.072	0.993	0.353	−0.003	0.008

Abbreviations: NLR, neutrophil to lymphocyte ratio; MLR, monocyte to lymphocyte ratio; C3 and C4, complement 3 and 4. Bold values indicate statistical significance (* *p* < 0.05).

## Data Availability

The datasets used and/or analyzed during the present study are available from the corresponding author.

## Supplementary Materials

The following supporting information can be downloaded at: https://www.mdpi.com/article/10.3390/jcm12113672/s1, Table S1: Comparative analysis of the ESSDAI domains between the two groups.
