# Supplementary material for: Comparative Analysis of Hematological and Immunological Parameters in Patients with Primary Sjögren’s Syndrome and Peripheral Neuropathy
Source: J Clin Med. 2023 May 25;12(11):3672. doi: 10.3390/jcm12113672 (PMC10254055; doi:10.3390/jcm12113672)
Supplement: Supplementary file 1 [file jcm-12-03672-s001.zip › jcm-2357462-supplementary.pdf]

Table S1. Comparative analysis of the ESSDAI domains between the two groups.

	PN+ Group	PN− Group	*p*-Value
	**No = 31 (25.61%)**	**No = 90 (74.38%)**	
Constitutional, *n* (%)	0	0	-
Lymphadenopathy, *n* (%)	5 (16.12%)	20 (22.22%)	0.328 [a]
Glandular, *n* (%)	15 (48.38%)	68 (75.55%)	**0.006** [a]
Articular, *n* (%)	19 (61.29%)	55 (61.11%)	0.581 [a]
Cutaneous, *n* (%)	12 (38.70%)	22 (24.44%)	0.471 [a]
Pulmonary, *n* (%)	10 (32.25%)	17 (18.88%)	0.138 [a]
Renal, *n* (%)	10 (32.25%)	13 (14.44%)	**0.031** [a]
Muscular, *n* (%)	4 (12.90%)	15 (16.66%)	0.429 [a]
Hematological, *n* (%)	13 (41.93%)	38 (42.22%)	0.575 [a]
Biological, *n* (%)	13 (41.93%)	19 (21.11%)	**0.023** [a]

Abbreviations: PN, peripheral neuropathy. [a] Chi- Square; statistical significance <0.05.
